# Association Between Living Alone and Physical Inactivity Among People With and Without Disability, Florida Behavioral Risk Factor Surveillance System, 2009

**DOI:** 10.5888/pcd11.140182

**Published:** 2014-10-09

**Authors:** César G. Escobar-Viera, Patrice D. Jones, Jessica R. Schumacher, Allyson G. Hall

**Affiliations:** Author Affiliations: Patrice D Jones, Allyson G Hall, Department of Health Services Research, Management and Policy, University of Florida, Gainesville, Florida; Jessica R Schumacher, Department of Population Health Sciences, University of Wisconsin, Madison, Wisconsin.

## Abstract

People with disability may be at risk of developing diseases due to physical inactivity; social support from family and friends is positively related to engaging in regular physical activity. We compared the association between living alone and engagement in physical activity among people with and without disability in Florida. We used multivariate logistical regression to analyze 2009 Florida Behavioral Risk Factor Surveillance System data (n = 10,902) to assess differences in physical activity in disability levels for respondents who lived alone versus those who did not. Respondents with a disability were less likely to engage in physical activity than were people without a disability, regardless of disability type, and the lowest rates of engaging in physical activity were found for people with disability who lived alone. Public health efforts should consider the role of household composition when targeting physical activity interventions among people with disability.

## Objective

People with disability may be at risk of developing diseases due to physical inactivity (1–3). The US Department of Health and Human Services recommends that people with disability engage in regular physical activities (4–6). Evidence indicates an inherent benefit of moderate to vigorous physical activity on health outcomes (7,8). Social support from family and friends is positively related to regular physical activity (9). Research is needed to understand whether people with disability are disproportionately affected by lack of support (2). We hypothesized that living alone will more negatively affect engagement in physical activity among people with disability in Florida than among those without disability.

## Methods

This cross-sectional study used 2009 Florida Behavioral Risk Factor Surveillance System (BRFSS) data. BRFSS is a random-digit–dial telephone household survey of adults aged 18 or older administered annually by state and territorial health agencies with support from the Centers for Disease Control and Prevention (CDC). BRFSS collects demographic and health-related data using core questions. In 2009, Florida added disability-related questions.

Respondents were asked BRFSS questions used to define disability, as well as state-added questions (Box). The state-added questions were used to create a 4-level disability severity variable: no disability and 3 mutually exclusive classifications for activity limitations for 6 months or longer and needing assistance with 1) instrumental activities of daily living (IADL) only; 2) assistance with activities of daily living (ADL), with or without IADL assistance; and 3) neither IADL nor ADL assistance. ADL are the basic tasks of self-care (eg, feeding, toileting, grooming, bathing, walking, putting on clothes oneself), and IADL are elaborate skills needed for successful independent living (ie, finance managing, handling transportation, shopping, preparing meals, using a telephone or other communication device, managing medications, doing housework, and doing basic maintenance). We constructed a dichotomous variable, with participants “living alone” if they had no adults and no children younger than 18 living in their household. Then we assessed CDC-defined physical activity including any, moderate, and vigorous activity for at least 10 minutes each time in a usual week (10).

**Box. Disability definition and state-added questions used to develop the categories of disability, Florida Behavioral Risk Factor Surveillance System (BRFSS), 2009.**

Questions used to define disability (BRFSS core questions)

Are you limited in any way in any activities because of physical, mental, or emotional problems?
Do you now have any health problem that requires you to use special equipment, such as a cane, a wheelchair, a special bed, or a special telephone?

Questions used to create the severity levels of disability (state-added questions)

Because of any impairment or health problem, do you need the help of others in handling your routine needs, such as everyday household chores, doing necessary business, shopping, or getting around for other purposes?
Because of any impairment or health problem, do you need the help of other persons with your personal care needs, such as eating, bathing, dressing, or getting around the house?
How long have your activities been limited due to this condition or impairment?

The response rate for the 2009 Florida BRFSS was 51% and included 12,055 respondents with landline telephones. Of these, 346 did not respond to questions that ascertained disability status; another 807 who reported activity limitations lasting less than 6 months were deemed to have a short-term disability and were excluded, yielding an analytic sample of 10,902.

We assessed demographic composition of the sample by age, sex, race, education, employment, income, marital status, and health behaviors (ie, smoking and drinking). Differences in physical activity measures for each disability classification were stratified by whether the respondent was living alone at the time of the survey and were assessed by using multivariate logistic regression analysis and considered significant at *P* < .05. Interactions between disability status and living alone were tested. To give model estimates meaningful interpretation, adjusted average predicted probabilities of physical activity for each disability classification were calculated and adjusted to the overall distribution of covariates. All analyses were conducted using Stata/IC12.1 for Windows (StataCorp, LP) by using weighting procedures to account for the complex sampling design of the BRFSS.

## Results

Overall, 51% of respondents were female, 61% were white, 60% were married, and 56% were employed. Mean age of respondents was 49 years, and 64% reported an annual household income below $50,000; 18% of the sample reported living alone. In total, 8,335 (76%) respondents had no disability, and of the remaining 2,567 who reported having a disability, 235 (9%) had ADL assistance needs, 713 (28%) had IADL assistance needs, and 1,619 (63%) had no needs. Respondents with a disability tended to be older, unmarried, and unemployed and had lower incomes than those with no disability.

We found a lower prevalence of participants engaging in each category of physical activity across all 3 levels of disability (Figure): any (running, gardening, or walking during the past month), moderate (bicycling, vacuuming, gardening, for at least 10 minutes per session in a usual week), and vigorous (running or heavy yard work, for at least 10 minutes per session in a usual week). One exception was found among people with ADL needs: a higher proportion engaged in vigorous activity than did people with activity limitations and IADL needs.

**Figure:**
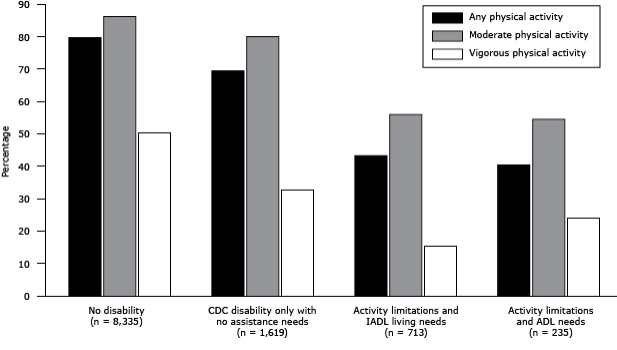
Proportion of Florida residents (N = 10,902) participating in any, moderate, or vigorous physical activity, by level of disability, Florida Behavioral Risk Factor Surveillance System, 2009. *Any* physical activity included running, gardening, or walking during the past month; *moderate* physical activity included bicycling, vacuuming, or gardening for at least 10 minutes per session in a usual week; and *vigorous* physical activity included running or heavy yard work for at least 10 minutes per session in a usual week. Abbreviations: ADL, activities of daily living; IADL, instrumental activities of daily living; CDC, Centers for Disease Control and Prevention **Table:** 

Disability/Physical Activity Level	Percentage
**No disability (n = 8,335)**	
Any	79.9
Moderate	86.4
Vigorous	50.7
**CDC-defined disability only with no assistance needs (n = 1,619)**	
Any	69.6
Moderate	80.2
Vigorous	32.8
**Activity limitations and IADL needs (n = 713)**	
Any	43.4
Moderate	56.1
Vigorous	15.5
**Activity limitations and ADL needs (n = 235)**	
Any	40.6
Moderate	54.6
Vigorous	24.2

The adjusted average predicted probabilities indicated that respondents who lived alone were less likely to engage in all levels of physical activity than were respondents who did not live alone (Table). Of respondents with activity limitations and ADL needs who did not live alone, the predicted probability of engaging in any physical activity was 52.3% (95% confidence interval [CI], 34.3–70.4), 52.8% (34.1–71.4) for moderate activity, and 23.8% (6.9–40.8) for vigorous activity. For the same subsample who lived alone, the probability of engaging in any physical activity was 47.2% (29.3–65.1), 41.6% (21.8–61.3) for moderate activity, and 15.0% (2.5–27.5) for vigorous activity. All comparisons were significant across regression models.

**Table:** Adjusted Average Predicted Probabilities of Any Physical Activity, Moderate Physical Activity, or Vigorous Physical Activity, by Disability Status and Whether Respondent Lives Alone, 2009 Florida Behavioral Risk Factor Surveillance System (N = 10,902)^a^

Disability/Living Status	Predicted Probability of Engaging in Physical Activity,^b^ % (95% CI)		
	Any (n = 4,439)	Moderate (n = 4,427)	Vigorous (n = 4,416)
**No disability**			
Does not live alone	72.9 (69.6–76.1)	82.6 (79.9–85.3)	44.8 (41.3–48.3)
Lives alone	67.9 (63.9–71.9)	75.3 (71.1–79.5)	32.4 (28.0–36.7)
**Disability only with no assistance needs**			
Does not live alone	69.5 (63.1–75.9)	81.3 (75.5–87.0)	35.7 (27.8–43.6)
Lives alone	64.5 (57.9–71.1)	73.6 (66.2–81.1)	24.9 (18.6–31.1)
**Activity limitations and IADL^c^ needs**			
Does not live alone	55.1 (43.2–67.0)	61.3 (49.1–73.6)	29.9 (18.5–41.2)
Lives alone	50.8 (39.0–62.6)	51.8 (38.1–65.6)	20.0 (11.2–28.7)
**Activity limitations and ADL^c^ needs**			
Does not live alone	52.3 (34.3–70.4)	52.8 (34.1–71.4)	23.8 (6.9–40.8)
Lives alone	47.2 (29.3–65.1)	41.6 (21.8–61.3)	15.0 (2.5–27.5)

Abbreviations: CI confidence interval; IADL, instrumental activities of daily living; ADL, activities of daily living.

a Models were adjusted for age, sex, race, education, employment, and income. All comparisons were significant (*P* < .05) in the logistic regression model.

b “Any” physical activity includes running, gardening, or walking during the previous month; “moderate” activity includes bicycling, vacuuming, or gardening for at least 10 min per session in a usual week; “vigorous” activity includes running or heavy yard work for at least 10 min per session in a usual week.

c ADL are the basic tasks of self-care (eg, feeding, toileting, grooming, bathing, walking, putting on clothes oneself), and IADL are elaborate skills needed for successful independent living (ie, finance managing, handling transportation, shopping, preparing meals, using a telephone or other communication device, managing medications, doing housework, and doing basic maintenance).

## Discussion

Our findings suggest that living alone is a predictor of engaging in physical activity, among people both with and without disability. Adjusted average predicted probabilities of engaging in physical activity showed a consistent low prevalence across disability levels, regardless of household composition. However, people who lived alone were less likely to engage in any type of physical activity than were those who did not live alone across disability levels, and this had a disproportional impact on people with disability. Thus, living alone was negatively associated with engaging in all levels of physical activity (any activity, moderate, or vigorous) for people with disability. Our finding that people with ADL needs reported engaging in more vigorous activity than did those with IADL needs is counterintuitive. We think these results reflect the more integral cognitive functions of people with disability with ADL needs, which allow them to seek sources of support. We believe this finding warrants further exploration.

Social support is important for engaging in healthy behaviors, including physical activity (7,8). Although living alone does not equate to lack of social network or support (11), it is associated with early onset of disability and death (1). Our findings could be used to develop a set of health interventions to promote wellness and combat social isolation, among people both with and without disabilities. For example, online support sites and Internet-based communities increase social support and sense of community among this population. Interventions that use these technologies (eg, mHealth) might have positive results engaging people with disability in physical activity (12).

Our study had limitations. We used data from a representative survey with a large sample size. Although results are generalizable to households with landlines in Florida, a steady decrease in the use of landlines among young people may have caused underrepresentation of this demographic group. Survey data were self-reported and are subject to recall and social desirability biases.

Public health efforts should consider the role of household composition in increasing physical activity, among people both with and without disability. Moreover, efforts in improving physical activity among people with disability should take into account social support, specifically household composition.
